# Serial bedside retinal optical coherence tomography angiography in sepsis associated circulatory dysfunction

**DOI:** 10.1186/s40635-026-00993-3

**Published:** 2026-10-27

**Authors:** André Rosa Alexandre, Gonçalo Meleiro Magalhães, Pedro Póvoa

**Affiliations:** 1 Intensive Care Department, Hospital da Luz Lisboa, Luz Saúde, Portugal; 2 NOVA Medical School, New University of Lisbon, Lisbon, Portugal; 3 Department of Critical Care Medicine, Hospital de São Francisco Xavier, ULS de Lisboa Ocidental EPE, Lisbon, Portugal; 4 Center for Clinical Epidemiology and Research Unit of Clinical Epidemiology, OUH Odense University Hospital, Odense, Denmark

## Dear editor,

Retinal optical coherence tomography angiography (OCTA) has emerged as a bedside technique for non-invasive assessment of the retinal microcirculation [1–3]. Whether serial OCTA can detect microvascular changes during vasopressor-treated sepsis remains uncertain.

We prospectively performed serial bedside OCTA in adults with sepsis and cardiovascular Sequential Organ Failure Assessment (SOFA) score ≥ 3 at Hospital da Luz Lisboa (February 2023–October 2024; NCT04593212). Funding constraints and termination of the device loan curtailed planned expansion to 165 patients. Of 56 screened, 30 were enrolled, 29 had valid baseline imaging and 13 underwent repeated examinations. Non-repetition followed vasopressor discontinuation (*n* = 14), technical difficulties (*n* = 1) or transfer (*n* = 1) (Supplementary Figure S1). Hyperlactataemia was not mandatory for enrolment. This analysis of patients with repeated examinations was exploratory. Four patients’ baseline examinations, but no subsequent measurements, were reported previously [3]. Baseline characteristics are summarised in Supplementary Table S1.

Patients were screened at diagnosis; enrolment and the first OCTA examination were required within 24 h of diagnosis. Subsequent examinations were scheduled every 24 h while patients received vasopressors. Superficial retinal scans (6 × 6 mm; Cirrus 6000 AngioPlex) required Signal Strength ≥ 7 and acceptable artefact screening. Image assessment was blinded to clinical information. Full-region perfusion density (PD), the perfused fraction of the entire Early Treatment Diabetic Retinopathy Study (ETDRS) grid (0–1), was selected before evaluating longitudinal results. Associations were analysed using binocular mean PD and a restricted-maximum-likelihood mixed model with study day as a fixed effect and a patient random intercept; uncertainty was assessed with 10,000 patient-level bootstrap resamples.

**Fig. 1:**
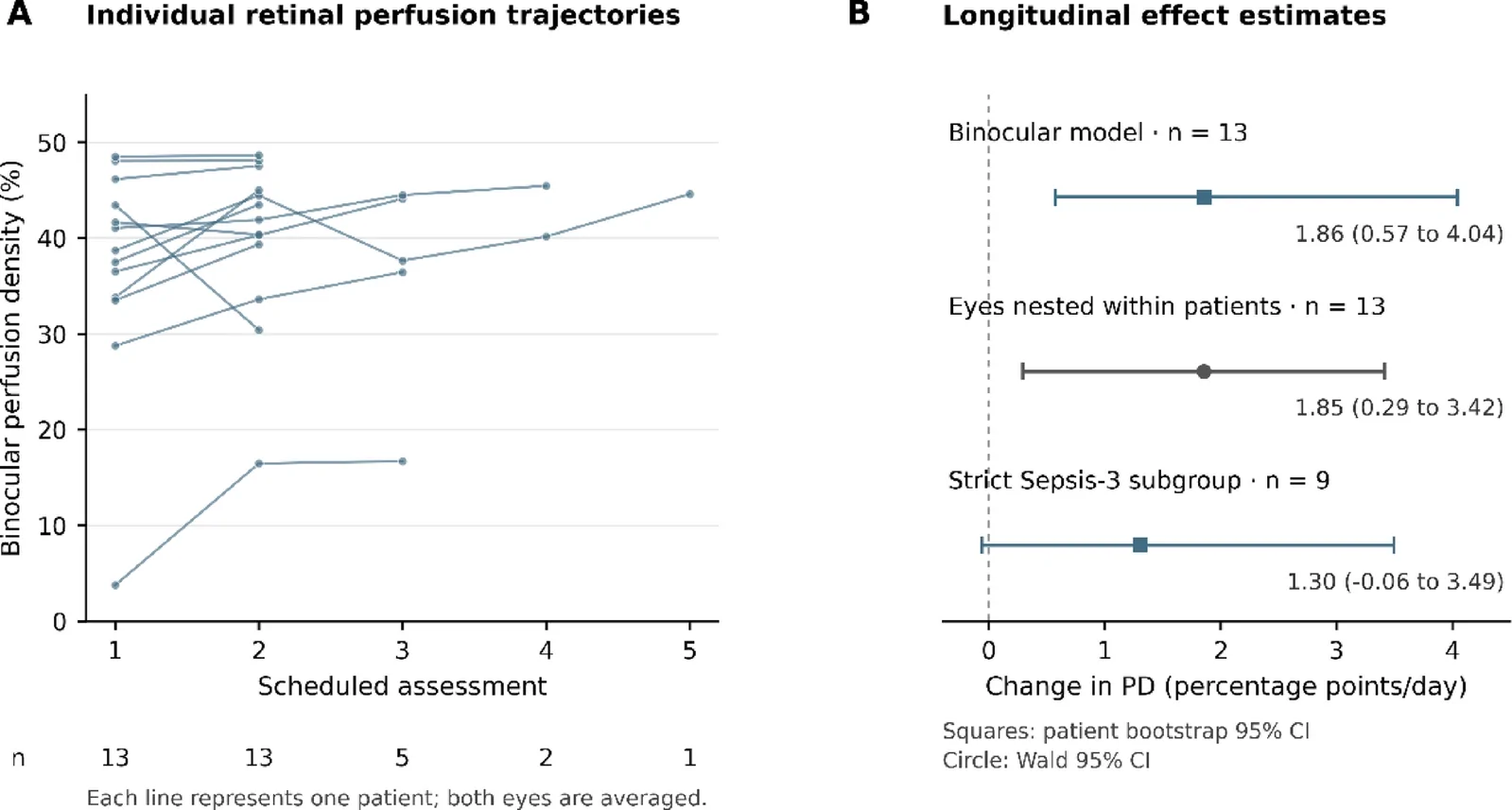
Serial retinal perfusion density and longitudinal effect estimates. (**A**) Observed mean binocular full-region retinal perfusion density (PD) at each scheduled assessment in 13 patients, comprising 34 examinations and 68 eye-level observations. Each line joins measurements from one patient; all patients receive the same visual emphasis. PD is expressed as a percentage of the total ETDRS grid area. Numbers below the axis indicate patients contributing at each assessment. Assessment 1 corresponds to the initial examination; subsequent assessments represent nominal daily visits during vasopressor treatment. (**B**) Estimated change in PD per nominal day from restricted-maximum-likelihood linear mixed-effects models. The main binocular model and strict Sepsis-3 subgroup model include a patient-specific random intercept; their intervals are 95% percentile confidence intervals from 10,000 patient-level bootstrap resamples retaining complete trajectories. The original eye-level model includes random intercepts for patients and eyes nested within patients and is shown with its asymptotic Wald 95% confidence interval. Labels report the number of patients, not eyes. Symbols denote point estimates; horizontal bars denote confidence intervals. The dashed line denotes no change. These exploratory estimates do not correct for informative discontinuation of follow-up

Thirteen patients generated 34 examinations and 68 eye-level observations, with 13, 13, 5, 2 and 1 patients at assessments 1 to 5, respectively (Fig. 1A; Supplementary Table S2). Baseline median PD was 38.7%. PD increased by 1.86% points per nominal day (Wald 95% CI 0.32–3.39; *p* = 0.018; bootstrap 95% CI 0.57–4.04). Between the first two examinations, PD increased in 11/13 patients, but the mean paired difference was imprecise (2.94% points; 95% CI − 0.92 to 6.80). Among nine patients meeting strict Sepsis-3 shock criteria [4], the bootstrap interval included zero (− 0.06 to 3.49% points/day; Fig. 1B).

Higher vasopressor score (VSI), calculated from mean weight-adjusted infusion rates derived from cumulative 24-hour doses [5], was associated with lower PD (β=−0.00143; *p* = 0.031). After mutual adjustment, neither study day nor VSI remained statistically significant (*p* = 0.298 and *p* = 0.491; Supplementary Table S3). Their inverse within-patient correlation (*r* = − 0.77) suggests collinearity; these findings do not establish a shared physiological mechanism.

This study has several limitations, including its small, single-centre sample, absence of formal reproducibility assessment and potential time-varying confounding. Follow-up was informative: rapidly recovering patients stopped contributing, whereas those requiring prolonged vasopressors remained under observation. Bootstrap resampling cannot correct this selection. Without a microcirculatory reference standard, changes in retinal PD cannot establish systemic microcirculatory recovery or prognostic value.

In conclusion, serial bedside retinal OCTA was feasible and PD was positively associated with observation time. Larger longitudinal studies are warranted to determine whether OCTA provides information beyond conventional haemodynamic monitoring or anticipates clinical recovery.

## Supplementary Information

Supplementary Material 1

## Data Availability

The datasets generated and/or analysed during the current study are not publicly available because they contain pseudonymized clinical and imaging data from critically ill patients that could potentially permit re-identification. The datasets are available from the corresponding author on reasonable request and, where applicable, subject to approval by the institutional Ethics Committee.
