# Supplementary material for: Serial bedside retinal optical coherence tomography angiography in sepsis associated circulatory dysfunction
Source: Intensive Care Med Exp. 2026 Oct 27;14:141. doi: 10.1186/s40635-026-00993-3 (PMC13713927; doi:10.1186/s40635-026-00993-3)
Supplement: Supplementary file 1 [file 40635_2026_993_MOESM1_ESM.docx]

Figure S1. Recruitment and selection of the longitudinal OCTA cohort. Of 56 screened patients, 30 were enrolled and 29 had at least one valid baseline eye. The patient without a valid baseline examination died before reassessment. Among the 29 patients with valid baseline imaging, 16 had no repeat examination for the reasons shown. Thirteen patients contributed 34 examinations and 68 eye-level observations. Healthy controls from the case–control phase are outside this longitudinal flow. Retinal exclusions included retinal disease or previous retinal surgery/photocoagulation. OCTA, optical coherence tomography angiography.

**Table S1 Baseline characteristics by availability of repeated OCTA**

| **Characteristic** | **Repeated OCTA (*n*=13)** | **No repeat OCTA (*n*=17)** |
| --- | --- | --- |
| Age, years | 75 [64–84] | 67 [47–81] |
| Male sex, *n*/N (%) | 6/13 (46.2) | 6/17 (35.3) |
| Diabetes mellitus, *n*/N (%) | 4/13 (30.8) | 3/17 (17.6) |
| Hypertension, *n*/N (%) | 6/13 (46.2) | 3/17 (17.6) |
| Source of infection, *n*/N (%) |  |  |
| Respiratory | 2/13 (15.4) | 1/17 (5.9) |
| Urinary | 4/13 (30.8) | 11/17 (64.7) |
| Skin and soft tissue | 1/13 (7.7) | 0/17 (0.0) |
| Intra-abdominal | 5/13 (38.5) | 4/17 (23.5) |
| Other | 1/13 (7.7) | 1/17 (5.9) |
| APACHE II score | 18 [13–20] | 18 [12–22] |
| SOFA score | 8 [7–10] | 7 [5–8] |
| Strict Sepsis-3 shock at screening, *n*/N (%) | 9/13 (69.2) | 14/17 (82.4) |
| Screening lactate, mmol/L | 2.6 [2.0–3.3] | 2.6 [2.3–3.5] |

Continuous variables are median [interquartile range]; categorical variables are *n*/N (%).

Screening lactate is distinguished from lactate measured at baseline OCTA. Strict Sepsis-3 shock refers to the screening assessment. OCTA, optical coherence tomography angiography; APACHE II, Acute Physiology and Chronic Health Evaluation II; SOFA, Sequential Organ Failure Assessment.

**Table S1 continued Baseline physiology and retinal imaging**

| **Characteristic** | **Repeated OCTA (*n*=13)** | **No repeat OCTA (*n*=17)** |
| --- | --- | --- |
| Temperature, °C | 36.9 [36.7–37.1] | 36.7 [36.2–36.8] |
| Heart rate, beats/min | 96 [87–100] | 90 [71–108] |
| Mean arterial pressure, mmHg | 72 [67–85] | 77 [71–85] |
| Capillary refill time, s | 2 [1–3] | 1 [1–2] |
| Lactate at baseline OCTA, mmol/L | 1.5 [1.1–2.4] | 1.6 [1.1–1.8] |
| Central venous oxygen saturation, % | 58 [54–67] | 60 [52–64] |
| Central venous-to-arterial CO₂ gap, mmHg | 8 [8–9] | 8 [6–9] |
| Haemoglobin, g/dL | 10.9 [10.4–12.4] | 9.9 [8.7–10.6] |
| PaCO₂, mmHg | 32 [28–34] | 34 [31–37] |
| Fluid balance, mL | +1933 [1279–2807] | +2007 [1034–2317] |
| Vasopressor score | 23.0 [12.9–38.0] | 17.2 [11.8–33.1] |
| Norepinephrine use, *n*/N (%) | 13/13 (100.0) | 17/17 (100.0) |
| Ventilatory support, *n*/N (%) | 8/13 (61.5) | 11/17 (64.7) |
| Full-region PD, % | 38.70 [33.80–43.45] | 43.90 [42.31–45.84]; *n*=16 |
| Full-region vessel density, mm/mm² | 16.3 [14.3–18.0] | 17.9 [17.2–18.8]; *n*=16 |
| Mean valid-eye Signal Strength | 9.5 [8.5–10.0] | 10.0 [9.5–10.0]; *n*=16 |
| ICU mortality, *n*/N (%) | 1/13 (7.7) | 1/17 (5.9) |

Continuous variables are median [interquartile range]; categorical variables are n/N (%). Baseline OCTA metrics were available for 29 patients; the patient without a valid baseline image died before repeat assessment. Retinal PD and vessel density are patient-level means across valid eyes (both eyes in the longitudinal cohort). PD is expressed as a percentage of the full ETDRS grid area. Fluid balance is reported as recorded in the baseline dataset.

Vasopressor score (VSI) was calculated from mean weight-adjusted infusion rates derived from cumulative 24-hour drug amounts, following Póvoa P et al. Crit Care. 2015;19:193. doi:10.1186/s13054-015-0921-x. These values do not represent the instantaneous infusion rate at OCTA.

PD, perfusion density; ETDRS, Early Treatment Diabetic Retinopathy Study; PaCO₂, arterial carbon dioxide partial pressure; ICU, intensive care unit.

**Table S2 Observed values at each scheduled OCTA assessment**

| **Variable** | **Assessment 1** | **Assessment 2** | **Assessment 3** | **Assessment 4** | **Assessment 5** |
| --- | --- | --- | --- | --- | --- |
| Patients, *n* | 13 | 13 | 5 | 2 | 1 |
| Eyes, *n* | 26 | 26 | 10 | 4 | 2 |
| Full-region binocular PD, % | 38.70 [33.80–43.45] | 41.90 [39.35–45.00] | 37.65 [36.45–44.10] | 40.15; 45.45 | 44.60 |
| Vasopressor score | 23.0 [12.9–38.0] | 9.6 [4.3–16.7] | 15.3 [3.6–16.1] | 10.9; 7.6 | 3.1 |
| Mean arterial pressure, mmHg | 72 [67–85] | 75 [71–78] | 78 [70–80] | 64; 107 | 64 |
| Lactate, mmol/L | 1.5 [1.1–2.4] | 1.0 [0.8–1.7] | 1.3 [1.1–2.1] | 0.7; 2.7 | 1.0 |
| SOFA score | 8 [7–10] | 6 [5–7] | 8 [7–8] | 6; 6 | 6 |

Assessments 1–3: median [interquartile range]. Assessment 4: individual values for two patients, in the same patient order for every row. Assessment 5: the observed value for one patient. Percentiles use linear interpolation. Assessment 1 is the initial OCTA; subsequent assessments represent scheduled daily examinations during vasopressor treatment. The changing sample composition precludes interpreting cross-sectional medians as a within-patient trajectory.

Each patient contributes one binocular mean PD and one clinical record per assessment. Vasopressor score uses mean weight-adjusted infusion rates derived from cumulative 24-hour drug amounts, rather than the instantaneous rate at OCTA.

SOFA, Sequential Organ Failure Assessment; PD, perfusion density.

**Table S3 Longitudinal models and sensitivity analyses**

| **Analysis** | ***n*** | **Records** | **Effect unit** | **Estimate** | **95% CI** | **CI method** | ***p*** |
| --- | --- | --- | --- | --- | --- | --- | --- |
| Main binocular model | 13 | 34 | PD pp/day | 1.86 | 0.57 to 4.04 | Bootstrap percentile | — |
| Main binocular model | 13 | 34 | PD pp/day | 1.86 | 0.32 to 3.39 | Wald | 0.018 |
| Original model with eyes nested in patients | 13 | 68 eyes | PD pp/day | 1.85 | 0.29 to 3.42 | Wald | 0.020 |
| Strict Sepsis-3 subgroup | 9 | 24 | PD pp/day | 1.30 | -0.06 to 3.49 | Bootstrap percentile | — |
| Excluding the ICU non-survivor | 12 | 31 | PD pp/day | 1.39 | -0.07 to 2.84 | Wald | 0.062 |
| Time adjusted for mean arterial pressure | 13 | 34 | PD pp/day | 1.83 | 0.24 to 3.42 | Wald | 0.024 |
| Time adjusted for lactate | 13 | 34 | PD pp/day | 1.82 | 0.17 to 3.47 | Wald | 0.031 |
| Time adjusted for SOFA | 13 | 34 | PD pp/day | 1.74 | -0.03 to 3.50 | Wald | 0.054 |
| Paired first–second assessment | 13 | 13 pairs | PD pp | 2.94 | -0.92 to 6.80 | Paired t | 0.123 |
| VSI alone: association with PD | 13 | 34 | PD pp/VSI point | -0.14 | -0.27 to -0.01 | Wald | 0.031 |
| Joint model: time coefficient | 13 | 34 | PD pp/day | 1.24 | -1.10 to 3.58 | Wald | 0.298 |
| Joint model: VSI coefficient | 13 | 34 | PD pp/VSI point | -0.07 | -0.26 to 0.12 | Wald | 0.491 |
| VSI change with time | 13 | 34 | VSI points/day | -8.11 | -11.38 to -4.84 | Wald | <0.001 |

*n* = patients; records = patient examinations unless otherwise stated. PD pp = perfusion-density percentage points. Daily effects use nominal study day, coded 0 at the initial examination. Mixed models were estimated by restricted maximum likelihood with a patient random intercept; the original eye-level model additionally includes an eye-within-patient random intercept. Wald intervals and *p* values use asymptotic normal inference. Bootstrap percentile intervals use 10,000 patient-level resamples retaining complete trajectories; no bootstrap *p* value is reported. Paired analysis uses a *t* interval with 12 degrees of freedom and is a difference between examinations, not a daily slope. All analyses are exploratory; no multiplicity adjustment was applied.

Time and VSI had a within-patient Pearson correlation of -0.767, calculated after centring both variables on each patient’s mean. Separate adjustment for MAP, lactate or SOFA does not address all potential time-varying confounding. Statistical implementation: Python with NumPy, pandas and SciPy.

Table S3 continued Leave one patient out sensitivity analysis

| **Patient omitted** | **Patients retained** | **Examinations retained** | **PD change pp/day** | **Wald 95% CI** | ***p*** |
| --- | --- | --- | --- | --- | --- |
| Patient 01 | 12 | 31 | 1.69 | 0.03 to 3.36 | 0.046 |
| Patient 02 | 12 | 32 | 1.88 | 0.30 to 3.47 | 0.020 |
| Patient 03 | 12 | 31 | 1.70 | 0.04 to 3.35 | 0.045 |
| Patient 04 | 12 | 32 | 1.91 | 0.33 to 3.50 | 0.018 |
| Patient 05 | 12 | 31 | 1.39 | -0.07 to 2.84 | 0.062 |
| Patient 06 | 12 | 29 | 2.60 | 0.61 to 4.60 | 0.010 |
| Patient 07 | 12 | 30 | 1.90 | 0.04 to 3.75 | 0.045 |
| Patient 08 | 12 | 32 | 1.93 | 0.35 to 3.51 | 0.016 |
| Patient 09 | 12 | 32 | 1.69 | 0.21 to 3.17 | 0.025 |
| Patient 10 | 12 | 32 | 2.19 | 0.92 to 3.46 | <0.001 |
| Patient 11 | 12 | 32 | 1.78 | 0.21 to 3.35 | 0.026 |
| Patient 12 | 12 | 32 | 1.78 | 0.21 to 3.35 | 0.027 |
| Patient 13 | 12 | 32 | 1.91 | 0.33 to 3.50 | 0.018 |

Patient labels are sequential figure-source labels, not original study identifiers. Each row refits the main binocular model after removing all observations from one patient. All estimated slopes remained positive. Confidence intervals and *p* values are asymptotic Wald estimates and are not adjusted for repeated sensitivity analyses. These analyses assess influence; they do not validate prognostic performance or resolve informative discontinuation.
